# Retinoid Receptors in Bone and Their Role in Bone Remodeling

**DOI:** 10.3389/fendo.2015.00031

**Published:** 2015-03-11

**Authors:** Petra Henning, H. Herschel Conaway, Ulf H. Lerner

**Affiliations:** ^1^Centre for Bone and Arthritis Research, Institute for Medicine, Sahlgrenska Academy, University of Gothenburg, Gothenburg, Sweden; ^2^Department of Physiology and Biophysics, University of Arkansas for Medical Sciences, Little Rock, AR, USA; ^3^Department of Molecular Periodontology, Umeå University, Umeå, Sweden

**Keywords:** vitamin A, retinoids, osteoclast, osteoblast, osteoporosis

## Abstract

Vitamin A (retinol) is a necessary and important constituent of the body which is provided by food intake of retinyl esters and carotenoids. Vitamin A is known best for being important for vision, but in addition to the eye, vitamin A is necessary in numerous other organs in the body, including the skeleton. Vitamin A is converted to an active compound, all-*trans*-retinoic acid (ATRA), which is responsible for most of its biological actions. ATRA binds to intracellular nuclear receptors called retinoic acid receptors (RARα, RARβ, RARγ). RARs and closely related retinoid X receptors (RXRα, RXRβ, RXRγ) form heterodimers which bind to DNA and function as ligand-activated transcription factors. It has been known for many years that hypervitaminosis A promotes skeleton fragility by increasing osteoclast formation and decreasing cortical bone mass. Some epidemiological studies have suggested that increased intake of vitamin A and increased serum levels of retinoids may decrease bone mineral density and increase fracture rate, but the literature on this is not conclusive. The current review summarizes how vitamin A is taken up by the intestine, metabolized, stored in the liver, and processed to ATRA. ATRA’s effects on formation and activity of osteoclasts and osteoblasts are outlined, and a summary of clinical data pertaining to vitamin A and bone is presented.

## Introduction

It was reported by Hopkins in 1906 that no animal can survive on a mixture of pure protein, fat, carbohydrates, water, and salt (1). Six years later, he reported that “accessory factors” present in astonishingly small amounts in milk support growth in rats (2). In 1918, McCollum suggested that “accessory fat soluble food factor” supporting growth should be called “fat soluble A” (3). Two years later, it was suggested by Drummond that “fat soluble A” should be called vitamin A (4). The chemical nature of vitamin A was described by Karrer in 1931 (5), but it was not until the end of the 1940s that vitamin A could be produced in large quantities (6, 7). Frederick Hopkins was awarded the Nobel Prize in 1929 for his work on vitamin A. Paul Karrer was awarded the Nobel Prize in 1937 for having established the chemical nature of many vitamins, including vitamin A. For a comprehensive background on the history of vitamin A, see Semba (8).

It was recognized in early studies that vitamin A is important for vision, and in 1933, Wald showed that the vitamin A derivative 11-*cis*-retinal makes up rhodopsin, together with the protein opsin (9). The visual signal transmitted from the retina to the central nervous system is caused by the light-energy-dependent decomposition of rhodopsin to opsin and all-*trans*-retinal. George Wald, together with Ragnar Granit and Haldan Keffer Hartline, received the Nobel Prize in 1967 for their discoveries concerning the primary physiological and chemical visual processes in the eye.

Vitamin A not only is important for growth and vision, but most cells in the body express vitamin A receptors. Important functions include stem cell differentiation, organ development and function, and the innate and acquired immune systems (10–16). In developing countries, vitamin A deficiency is common, and vitamin A supplementation can save countless lives at a minimal cost (17). Vitamin A is used for treatment of skin disorders like acne and also for different malignant tumors, particularly acute promyelocytic leukemia. It also is used for Kaposi’s sarcoma, head and neck squamous cell carcinoma, ovarian carcinoma, and neuroblastoma (18, 19).

Pathological changes in the outer cortex of bone characteristic of hypervitaminosis A have been observed in the tibial shaft from a partial *Homo erectus* skeleton found in Kenya (20). Early explorers suffered from vitamin A intoxication after consumption of polar bear liver, which caused vertigo, vomiting, diarrhea, headache, convulsions, peeling of the skin, and sometimes death (21–23). In experimentally induced hypervitaminosis A in animals, it was observed as early as the 1920s that excessive vitamin A results in thinning of the cortex of long bones and in spontaneous fractures (24). In epidemiological studies, it has been shown that increased levels of vitamin A in serum can be associated with decreased bone mass and increased risk for fractures (25, 26).

It is the aim of the present review to describe both the presence and function of vitamin A receptors in bone and to summarize the current knowledge of clinical studies investigating the role of vitamin A for bone mass and fracture risk.

## Vitamin A Uptake and Metabolism

**Figure 1:**
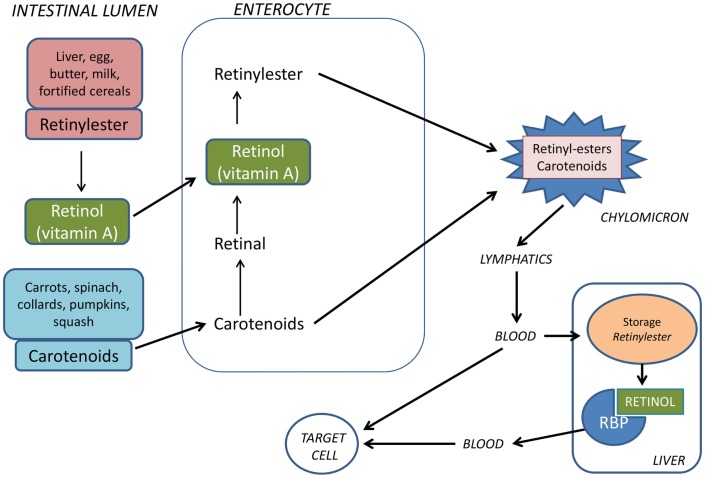
**Vitamin A is provided from the food either as preformed vitamin A (retinyl esters) or as provitamin A carotenoids**. Retinyl esters are hydrolyzed by pancreatic and intestinal enzymes and free retinol is taken up by the enterocytes. Half of the carotenoids is oxidized to retinal and then reduced to retinol. Retinol is esterified with long-chain fatty acids and incorporated into chylomicrons together with intact carotenoids and then carried by the lymphatics. The chylomicrons are taken up by hepatocytes in the liver where vitamin A is stored as retinyl esters. Before being released from the liver to the circulation, retinyl esters are hydrolyzed to retinol which binds to retinol-binding protein (RBP).

Vitamin A is obtained from the diet either as retinyl esters in eggs, liver, bottled milk or fortified cereals, or as carotenoids (e.g., β-carotene) in vegetables such as carrots or spinach. Approximately, 75% of vitamin A comes from retinyl esters. Retinyl esters and carotenoids taken up by enterocytes are incorporated in chylomicrons (Figure 1). These are transported by the lymphatics and then released into the circulatory system. Approximately, 66–75% of dietary retinoid is eventually taken up by hepatocytes, where vitamin A can be stored as retinyl esters or hydrolyzed to retinol, which binds to retinol-binding protein (RBP) before being released into the bloodstream (27). The remaining dietary retinoids are taken up by extra-hepatic tissues such as white adipose tissue, skeletal muscle, heart, lungs, and kidneys (28).

**Figure 2:**
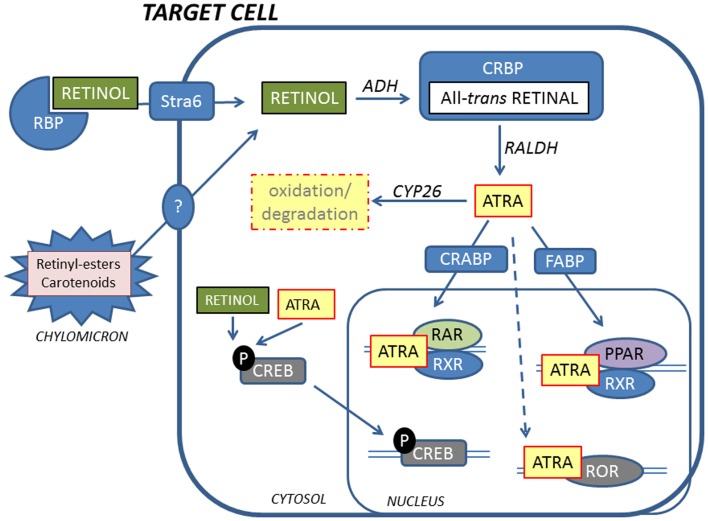
**Retinoids reach target cells mainly in the form of retinol bound to RBP**. A fraction of retinoids is also delivered by chylomicrons. Inside the cell, retinol is oxidized to the active metabolite ATRA by ADH and RALDH via all-*trans*-retinal that is bound by CRBP. ATRA is shuttled to the nucleus by CRABP and FABP, facilitating binding to RARs and PPARs, respectively. RARs and PPARs form heterodimers with RXRs to activate transcription. In addition, ATRA can bind to RORs to initiate transcription. Non-genomic effects of retinoids include phosphorylation of CREB that translocates to the nucleus and activates genes. ATRA is inactivated by oxidation by CYP26 enzymes.

Retinoid in the form of all-*trans* retinol is transported from the liver to peripheral cells bound to RBP in plasma. In the fasting state, >95% of retinoid in the circulation is found as retinol bound to RBP. Approximately, 25–33% of dietary retinoid that is absorbed in the intestine is delivered to tissues other than the liver by chylomicrons (27). A transmembrane-spanning receptor stimulated by retinoic acid receptor (STRA6) mediates the cellular uptake of retinol from RBP, while hydrolysis of retinyl esters by lipoprotein lipase is thought to facilitate uptake of retinol from chylomicrons (29, 30) (Figure 2). Carotenoids associated with lipoproteins in chylomicrons can be taken up by lipoprotein-specific receptors and converted to retinaldehyde (RALD) by β-caroten-15,15′-monooxygenase (BCMO1) (31). Bone is the second most important organ for clearance of chylomicron remnants, and it has been reported that other fat soluble vitamins can be delivered to osteoblasts *in vivo* via chylomicrons (32). Additionally, the active metabolite all-*trans*-retinoic acid (ATRA) is present at low levels in serum bound to albumin and has been shown to contribute to tissue levels of ATRA (33).

Inside target cells, retinol is oxidized to retinal by alcohol dehydrogenases and bound to cellular retinol-binding protein (CRBP). Retinal is then oxidized to ATRA, the biologically active metabolite of vitamin A, by retinal dehydrogenases (RALDH). Cellular levels of ATRA are regulated by the balance between synthesis by RALDH and oxidative metabolism by cytochrome P450s such as CYP26A1 and CYP26B1 (34–37). ATRA is bound to cellular retinoic acid-binding proteins (CRABP) and directed to the nucleus for activation of specific nuclear receptors.

## Retinoid Receptors

Retinoids activate and repress expression of genes by both genomic and non-genomic mechanisms. Two families of nuclear receptors, retinoic acid receptors (RARs) and retinoid X receptors (RXRs), are the primary receptors and mediators of retinoid effects (38, 39). Each receptor family is made up of three isotypes (α, β, and γ), produced by separate genes. In addition, alternative splicing and different promoter usage give rise to at least two different isoforms for each isotype (38). While the gene sequence for each of the RAR isotypes differs significantly from the other two, the sequences for each isotype are highly conserved between humans and mice, leading to the speculation that each RAR isotype has a specific function (40). RARs dimerize with RXRs, and the heterodimers function as transcription factors, activating retinoic acid response elements (RAREs) in the promoter regions of target genes. Most retinol signaling in cells is thought to be mediated by ATRA-binding RAR in RAR/RXR heterodimers (38, 41). The binding of ATRA to the RAR/RXR complex induces a conformational change in the ligand-binding domain of the receptor, which facilitates the recruitment of coactivators, such as members of the steroid receptor co-activator (SRC)/p160 family and p300/ CREB-binding protein (CBP) (38, 39). In the absence of ligand, the coactivators are replaced by corepressors, such as nuclear receptor corepressor (NCoR), silencing mediator of RAR and thyroid hormone receptor (SMRT), mSin3A, and histone deacetylases (HDACs), resulting in active repression of transcription of target genes by RAR/RXR heterodimers (38, 42, 43).

Cellular retinoic acid-binding protein II (CRABPII) shuttles ATRA to the nucleus and facilitates binding of RARs. ATRA is also shuttled to the nucleus by the fatty acid-binding protein (FABP)5. FABP, in contrast to CRABPII, facilitates binding of ATRA to peroxisome proliferator-activated receptors (PPARs), α, β/δ, and γ, which is another group of nuclear receptors that forms heterodimers with RXR (39, 44–47). PPAR/RXR heterodimers function as transcription factors, activating PPAR response elements (PPRE) in target genes. It has been hypothesized that ATRA can have opposing effects depending upon CRABPII/RAR or FABP5/PPAR β/δ binding in keratinocytes and carcinomas, but this has so far not been tested in bone cells (44, 45).

In addition to RARs, RXRs, and PPARs, retinoids can bind retinoid-related orphan receptors (ROR) β and γ (48, 49). RORs do not form heterodimers with RXR but regulate gene transcription by binding as monomers to specific ROR response elements (ROREs) in target genes (50, 51). RORβ has been shown to suppress mineralization and to decrease expression of *Bglap* (encoding osteocalcin) and *Sp7* (encoding osterix) mRNA in cultured murine osteoblasts (52). RORα has been shown to be involved in osteoblast metabolism and RORα-deficient mice have abnormal bone development (53, 54).

In addition to the genomic signaling via ATRA binding to different nuclear receptors that regulate RAREs, PPREs, and ROREs in target genes, retinoids can have rapid non-genomic/non-classical actions as well. ATRA induces a rapid phosphorylation of cyclic AMP response element-binding protein (CREB), which translocates to the nucleus, binds, and activates genes containing cyclic AMP response elements (CRE) in their promoters (39, 55). This effect is not limited to ATRA, but also can be exerted by retinol, and does not involve RARs (56). Another type of non-genomic effect has been described for cytosolic RARα. In neuronal cells, RARα has been shown to act as a RNA-binding protein that associates with a subset of mRNAs and inhibits their translation (57–59).

## Effects by Retinoids on Bone Resorption

### Effects by vitamin A on bone resorption *in vivo*

There are only a limited number of experimental studies investigating the effect by vitamin A on the skeleton. Trechsel et al. reported that treatment of rats with the retinoid Ro 13-6298 rapidly (2–4 days) caused hypercalcemia and decreased bone mass, responses associated with an enhanced number of periosteal osteoclasts (60). The fact that bisphosphonate decreased the effect by the retinoid on bone mass suggests that at least part of the effect was caused by enhanced osteoclast formation. Similarly, it was reported by Hough et al. that a high dose of retinyl palmitate enhanced osteoclast numbers and increased urinary secretion of hydroxyproline in rats (61).

**Figure 3:**
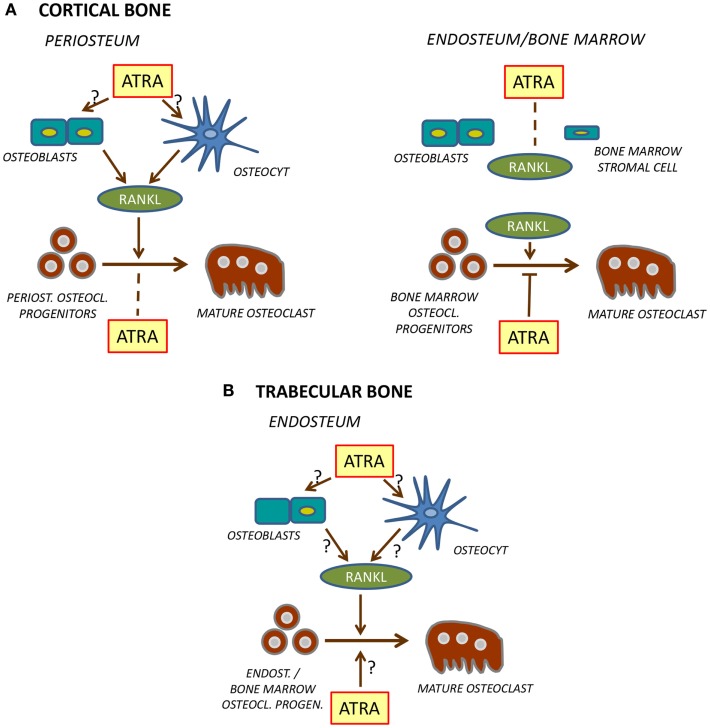
**Regulation of osteoclast formation in cortical (A) and trabecular (B) bone**. At the periosteal site of cortical bone [**(A)**, *left*], ATRA stimulates RANKL production in osteoblasts and/or osteocytes which leads to stimulation of differentiation of mature osteoclasts from osteoclast progenitors. Unlike in bone marrow, ATRA does not inhibit differentiation of these osteoclast progenitors. In bone marrow or at endosteal site [**(A)**, *right*], ATRA does not stimulate RANKL formation but inhibits differentiation of osteoclast progenitors to mature osteoclasts. The role of ATRA for osteoclast formation on the endosteal surfaces of trabecular bone **(B)** is currently not known.

More detailed studies have been performed by Kneissel et al. (62) and by Lind et al. (63), both demonstrating decreased bone mass and enhanced osteoclast formation by hypervitaminosis A. Treatment of male or female rats with either Ro 13-6298 for 4 days (62), or with a mixture of retinyl palmitate/retinyl acetate for 7 days (63), resulted in decreased cortical bone mass and an enhanced number of osteoclasts at the periosteal side of cortical bone, responses decreased by alendronate (Figure 3). Kneissel et al., however, found no effect on trabecular bone mass even though the number of trabecular osteoclasts was decreased, whereas Lind et al. observed decreased trabecular bone mass but with no effect on osteoclast number (Figure 3). These divergent findings are difficult to explain, but might be influenced by effects on bone formation by vitamin A since this was not assessed.

In the study by Kneissel et al., circulating levels of the osteoclastic resorption marker tartrate-resistant acid phosphatase 5b (TRAP5b) was increased, whereas Lind et al. reported decreased levels of circulating TRAP5b, carboxy-terminal collagen crosslinks (released to circulation during degradation of bone matrix) and osteoclastogenic cytokine receptor activator of nuclear factor κB ligand (RANKL). The increased TRAP5b is consistent with increased periosteal osteoclast numbers and decreased bone mass. Decreased TRAP5b, RANKL, and bone matrix fragments, though, should indicate decreased osteoclast numbers, which is in contrast to the enhanced number of periosteal osteoclasts reported by Lind et al. These authors also noted a substantial reduction of osteoclast numbers at the endosteal side of cortical bone. It might be that this response was more dominating than the periosteal response, and that the biochemical markers reflected the inhibition of osteoclastogenesis at the endosteal site. Mechanistically, Lind et al. suggest that decreased numbers of endosteal osteoclasts might be due to hypoxia caused by reduction of blood vessels in the bone marrow close to the endosteal surfaces. Interestingly, hypoxia-related genes, such as *Hif1a* (hypoxia-inducible factor 1 alpha) and the downstream genes *Twist1* (twist family basic helix-loop-helix transcription factor 1) and *Mmp2* (matrix metallopeptidase 2), also were decreased by hypervitaminosis A in the bone marrow. It is clear that hypervitaminosis A results in decreased cortical bone mass associated with increased numbers of periosteal osteoclasts (Figure 3). The effect is large enough to cause decreased strength as shown by three-point bending (63). More detailed studies are needed, however, to assess osteoclast formation on bone surfaces facing bone marrow. Such studies should include treatment with different concentrations of vitamin A and assessment at different time points after treatment and at different ages of experimental animals. Recently, it was reported (64) that both cortical bone mass and bone size in response to treatment with retinyl palmitate was different in young (2–3 months), middle-aged (8–10 months), and old (18–20 months) rats, but no studies at the cellular level were performed.

### Effects by vitamin A on bone resorption in organ culture

Increased numbers of osteoclasts as a consequence of hypervitaminosis A was reported as early as 1934 by Strauss and Maddock (24). That this was a direct effect by vitamin A was shown by Barnicot (65) using fragments of crystalline vitamin A acetate applied on parietal bones transplanted to mice brains and by Fell and Mellanby (66) after adding plasma from fowl treated with high doses of vitamin A to organ-cultured chicken or mouse long bones. The decreased amounts of bone were not always associated with an increased number of osteoclasts, and it was speculated that other cells may be responsible for the action of vitamin A. Raisz showed that vitamin A increased bone resorption in organ-cultured, newborn mouse parietal bones and that, in parallel, the number of osteoclasts was enhanced, although the number was less than that induced by parathyroid hormone (PTH), prompting the suggestion that vitamin A caused bone resorption primarily by increasing lysosome enzyme release, rather than by increasing osteoclast formation (67). Later, Raisz et al. demonstrated the importance of osteoclastogenesis for the effect of vitamin A on bone resorption by showing that no effect could be obtained in bone organ cultures from osteopetrotic *mi/mi* mice, which are unable to form osteoclasts due to mutation in the *Mitf* gene *(microphthalmia-associated transcription factor f)* (68). Since then, several groups have reported that both retinol and ATRA stimulate bone resorption in different organ-culture models (61, 69–72). Recently, we showed that ATRA-stimulated bone resorption and the number of cathepsin K^+^ mature osteoclasts in organ-cultured, neonatal mouse calvarial bones (73). The ATRA response was abolished by calcitonin and zoledronic acid and was associated with increased expression of osteoclastic genes, such as those encoding calcitonin receptor, TRAP, and cathepsin K. These experiments demonstrate that the bone-resorptive effect by ATRA in organ-cultured bone is dependent upon differentiation and the activity of osteoclasts.

The observations in bone organ culture consistently have shown that vitamin A can stimulate bone resorption and osteoclast formation. We have shown that the bone-resorptive effect in such organ cultures is dependent upon the osteoclastogenic cytokine RANKL (73). Thus, ATRA increased the mRNA and protein expression of RANKL and transiently decreased the RANKL inhibitor osteoprotegerin (OPG) at the mRNA level with no effect on OPG protein. The effect on bone resorption and osteoclastic genes was inhibited by exogenously added OPG, demonstrating the crucial role of RANKL for the stimulatory effect by ATRA on osteoclastogenesis and bone resorption. Nonetheless, it was not demonstrated which cell type responded to vitamin A with enhanced RANKL.

By using a pharmacological approach with a variety of different agonists for the different RARs, we provided evidence that RARα is mediating the stimulatory effects by ATRA on RANKL and bone resorption (73).

Thus, organ-culture studies are in agreement with *in vivo* studies showing increased formation of periosteal osteoclasts, although it remains to be definitively proven which cell type in bone is the primary target for vitamin A (Figure 3). Interestingly, it has recently been reported that treatment of the osteoblastic cell line MC3T3-E1 with ATRA for 2–3 weeks up-regulates *Tnfsf11* mRNA (encoding RANKL) while in parallel decreasing osteoblast differentiation (74).

### Effects by vitamin A on osteoclast formation in cell cultures

Experiments in organ-cultured bones suggest that vitamin A stimulates osteoclastogenesis by increasing the differentiation of mononuclear progenitors present in periosteum/endosteum by enhancing periosteal/endosteal RANKL, which is in agreement with observations made at cortical periosteal surfaces *in vivo* in murine animal models. Osteoclasts, though, are also formed on surfaces of bone facing bone marrow, with divergent effects by vitamin A observed in *in vivo* studies. Therefore, it has been of interest to assess whether vitamin A, similar to PTH and 1,25(OH)_2_-vitamin D3, can stimulate osteoclast formation in bone marrow cultures containing bone marrow stromal cells and hematopoietic cells, including osteoclast progenitors.

Using bone marrow cell cultures from human ribs, it has been found that, in contrast to 1,25(OH)_2_-vitamin D3, ATRA had no stimulatory effect on osteoclast formation (75). A similar observation has been made using rat bone marrow cells (71). Recently, we reported that, whereas PTH and 1,25(OH)_2_-vitamin D3 stimulate osteoclast formation in mouse bone marrow cultures, ATRA had no effect (76). Nor did ATRA induce expression of osteoclastic genes, such as those encoding calcitonin receptor, TRAP, and cathepsin K, or osteoclastogenic genes like nuclear factor of activated T cells 1 (*Nfatc1*) and FBJ osteosarcoma oncogene (*Fos*, c-Fos). The explanation for this is that, unlike 1,25(OH)_2_-vitmain D3 or PTH, ATRA does not induce mRNA expression of *Tnfsf11* (encoding RANKL). It currently is not known if the lack of effect on *Tnfsf11*expression in bone marrow stromal cells is due to the absence of retinoid receptors in these cells, or if stromal cell retinoid receptors are unable to induce the *Tnfsf11*gene in such cells. It is apparent, though, that bone marrow stromal cells are different from calvarial bone cells in terms of responsiveness to retinoids.

Unexpectedly, retinoids have been found to inhibit 1,25(OH)_2_-vitamin D3-stimulated osteoclast formation in rat bone marrow cultures (77) and in co-cultures containing mouse bone marrow cells and mouse calvarial osteoblasts (78). We have reported that ATRA inhibits osteoclast formation in mouse bone marrow cultures stimulated by either 1,25(OH)_2_-vitamin D3 or PTH (76). ATRA did not affect 1,25(OH)_2_-vitamin D3-induced expression of *Tnfsf11* mRNA or down-regulation of *Tnfrsf11b* mRNA (encoding OPG), showing that ATRA did not affect 1,25(OH)_2_-vitamin D3 signaling in stromal cells. The fact that ATRA inhibited hormone-induced up-regulation of osteoclastic genes indicates that ATRA inhibits osteoclast progenitor cell differentiation, rather than acting at a later step during osteoclastogenesis. Similar to the findings in hormone-stimulated bone marrow cultures, ATRA inhibits RANKL-induced osteoclast formation in mouse spleen cell cultures (76). It is, however, not possible to conclude from the crude bone marrow and spleen cell cultures that ATRA acts directly on osteoclast progenitor cells since other cells present in these cultures might respond to ATRA by secreting osteoclast inhibitory factor(s).

Conclusive evidence that vitamin A can act directly on osteoclast progenitor has been obtained in cells purified from mouse bone marrow and human blood. Using RANKL-stimulated, non-adherent monocytes/macrophage from mouse bone marrow, Kneissel et al. (62) reported that ATRA inhibited osteoclast formation. Using highly purified macrophage colony-stimulating factor (M-CSF)-expanded macrophages from mouse bone marrow, we found that ATRA abolished RANKL-stimulated osteoclast formation with a half maximal effect at 0.3 nM (76). To obtain maximal inhibition, ATRA had to be added along with RANKL, but not after RANKL addition, and withdrawal of ATRA 6 h after adding RANKL and ATRA together still resulted in strong inhibition. These observations suggest that ATRA interferes at an early interval of RANKL stimulation. Further evidence for this was the finding that ATRA strongly inhibited RANKL-induced mRNA expression of the osteoclastic genes *Calcr, Ctsk*, and *Acp5* (encoding calcitonin receptor, cathepsin K, and TRAP, respectively), with half maximal inhibition at 0.3 nM. Furthermore, the macrophage transcription factor *Mafb*, which is down-regulated by RANKL during osteoclastogenesis, still continued to be highly expressed after ATRA treatment, indicating that the cells were arrested at the macrophage state.

Similar to the observation in mouse osteoclast progenitor cell cultures, it has been reported that ATRA abolishes osteoclast formation in RANKL-stimulated, highly purified CD14^+^ monocytes from human peripheral blood, with inhibition observed at and above 0.04 nM of ATRA (79). Furthermore, the macrophage transcription factor interferon regulatory factor-8 (*IRF-8*) was not down-regulated by ATRA treatment, indicating that these cells were arrested at the monocyte/macrophage state. RANKL-induced osteoclast differentiation is dependent on a variety of kinases and transcription factors, which are regulated both at the transcriptional and activation levels (80–82). Three important transcription factors are activator protein-1 (AP-1), nuclear factor kappa B (NF-κB), and NFATc1, the latter being regarded as the master regulator of osteoclastogenesis (83). Thus, RANKL induces an early and prolonged mRNA expression of the AP-1 subunit *Fos* (encoding c-Fos), an early and transient induction of the canonical NF-κB subunits *Nfkb1 (p105/p50)* and *Rela* (*p65)*, an early and prolonged induction of the non-canonical NF-κB subunits *Nfkb2 (p100/p52)* and *Relb*, and a delayed up-regulation of *Nfatc1*. In contrast, ATRA inhibits the RANKL-induced, increased mRNA expression of *Fos, Nfkb2, Relb*, and *Nfatc1* in the purified mouse bone marrow macrophages, again with half maximal inhibition at 0.3 nM (76). It was further shown that ATRA also inhibited the RANKL-induced protein expression of c-Fos and Nfatc1. These observations indicate that ATRA inhibits signals which are downstream from the receptor activated nuclear factor kappa B (RANK) receptor, providing an explanation for why ATRA inhibits osteoclastogenesis in progenitors from mouse bone marrow. It also was found that ATRA inhibited the mRNA expression of *Tnfrsf11a* (encoding RANK), but not that of the M-CSF receptor *Csf1r* (colony-stimulating factor 1 receptor). However, *Tnfrsf11a* mRNA was decreased substantially later than the mRNA expression of *Fos* and *Nfatc1*, indicating that decreased *Tnfrsf11a* expression is not the primary event by which ATRA inhibits osteoclastogenesis.

Mouse bone marrow macrophages abundantly express *Rara* mRNA, but express less *Rarb* and *Rarg* mRNA (76). At the protein level, RARα and RARβ were similarly expressed, whereas hardly any cells expressed RARγ, as assessed by flow cytometry. The inhibitory effect by ATRA on osteoclastogenesis was shared by 9-*cis* retinoic acid and TTNPB, which, like ATRA, activate all three RARs. More importantly, the effect was shared also by the RARα-specific agonist GR104, and the inhibition by ATRA was decreased by the RARα antagonist GR110. These findings, together with the observations that the RARγ agonist A7980 and the RARβ/γ agonist GR103 were considerably less potent inhibitors of RANKL-induced osteoclastogenesis in the bone marrow macrophage cultures, suggest that RARα is the most important RAR-mediating retinoid-induced inhibitor of osteoclastogenesis.

As discussed previously, ATRA may exert biological effects not only by RARs but also via activation of PPARβ/δ (44–46). Crucial for this to occur is the intracellular binding of ATRA to FABP5, which leads to shuttling of ATRA to PPARβ/δ. Since we have observed that the mouse osteoclast progenitors express *Fabp5* mRNA, we evaluated if osteoclast inhibition might also be mediated by activation of PPARβ/δ. GW072, however, which is an activator of this receptor, did not inhibit RANKL-induced osteoclastogenesis, but instead potentiated the formation of numerous, oversized osteoclasts (76).

It seems clear that retinoids stimulate periosteal osteoclast formation both *in vivo* and in organ-cultured murine bones through enhanced RANKL expression, whereas RANKL-induced osteoclastogenesis in mouse bone marrow macrophage or human peripheral monocytes cultures is inhibited (Figure 3). The reason osteoclast progenitors in periosteal bone are not sensitive to retinoid inhibition is currently unclear but might be because osteoclast progenitors at different sites are heterogeneous, perhaps some lacking expression of retinoid receptors in periosteal osteoclasts but not in bone marrow or peripheral blood progenitors. Another possibility might be that some cells in the periosteum release molecules, making the periosteal osteoclast progenitors resistant to retinoids. Further experiments are needed to explore these possibilities.

### Effects by vitamin A on mature osteoclasts

All-*trans*-retinoic acid has been reported to increase mature osteoclast activity of rabbit osteoclasts on dentin slices (84) and 9-*cis* retinoic acid to stimulate mature rat osteoclasts on bovine cortical bone (85). Nevertheless, bone-resorbing activity of chicken osteoclasts on either bovine cortical bone slices or on sperm whale dentin was found to be inhibited by ATRA (86). These studies indicate that mature osteoclasts express retinoid receptors, but due to the divergent findings, it is currently not possible to make precise determinations about how these receptors might be linked to the bone-resorbing activity of these cells.

## Effects by Retinoids on Bone Formation

### Effects by vitamin A on bone formation *in vivo*

**Figure 4:**
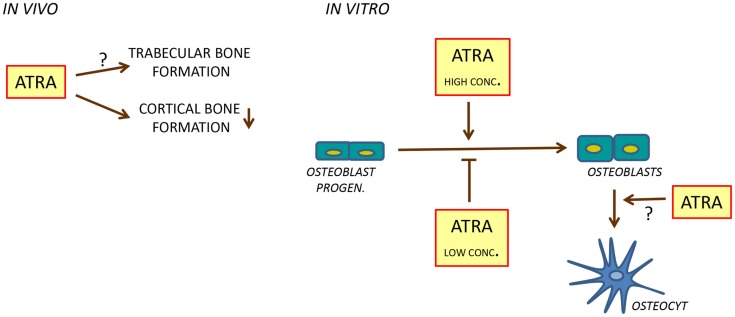
**Regulation of bone formation by ATRA**. In rats, ATRA inhibits bone formation in cortical bone (*left*). In cell cultures, ATRA seems to inhibit osteoblast differentiation at low concentrations and to stimulate at high concentrations (*right*). In addition, ATRA may stimulate differentiation of osteoblasts to osteocytes.

Hypervitaminosis A in rats, caused by a mixture of retinyl palmitate and retinyl acetate in pellets, has been found to result in osteocyte-rich woven bone along endosteal bone surfaces in long bones, which together with the observations that increased immunohistochemical staining of osteocalcin is increased at endosteal surfaces, and that the mRNA expression of osteoblastic genes such as *Alpl* (encoding alkaline phosphatase) and the transcription factor *Runx2* in the bone marrow is enhanced, suggest that vitamin A may have stimulatory effect on bone formation (63). However, using dynamic histomorphometry, a more accurate measurement of bone formation *in vivo*, it has recently been shown that rats fed with this diet exhibit decreased mineralizing surfaces, bone formation, and mineralized apposition rate in cortical bone (74) (Figure 4). Similar data has been reported by Kneissel et al. in rats treated with the retinol Ro 13-6298 (62). In the latter study, the effect seemed to be specific for cortical bone since no effects on primary and secondary spongiosa were observed.

In contrast to these observations, it has been found that lack of ATRA due to deficiency of RALDH (encoded by the gene *Aldh1a1*), the rate limiting enzyme in ATRA biosynthesis results in increased bone mass (87). In these studies, *Aldh1a1*-deficient female mice were compared to age- and sex-matched C57BL/6 mice. Increased bone density was observed in young (12 weeks) and aged (36 weeks) female mice. Mice with *Aldh1a1* deficiency exhibited increased cortical and trabecular bone mass as assessed by microCT. Using histomorphometry, thicker cortical bones was observed, whereas no significant changes of trabecular bone were found, indicating that RALDH mainly affects cortical bone. On the other hand, the histomorphometric analyses did not demonstrate significant changes in the number of osteoblasts or osteoclasts, nor did dynamic histomorphometry show any effects on bone formation. Therefore, it is difficult to understand the mechanisms causing increased bone mass in these mice.

Some observations were made, however, that indicate increased osteoblastic activity might be involved. Bone marrow adiposity was clearly enhanced, suggesting that RALDH may affect bone marrow stromal cell differentiation. Interestingly, bone marrow cells from *Aldh1a1*-deficient mice were more prone to undergo both osteoblastic and adipogenic differentiation, which seemed to be due to increased expression of bone morphogenetic protein 2 (BMP2). These effects might be due to lack of ATRA, but also could be caused by accumulation of RALD which was not being converted to ATRA. In agreement with this, addition of RALD to bone marrow stromal cells resulted in increased BMP2 independent of its conversion to ATRA, but dependent on retinoic receptors. Further studies are needed to gain more insight into the effect of retinoids on bone formation *in vivo* in different parts of the skeleton.

### Effects by vitamin A on osteoblast cultures

Cell culture studies using different osteoblastic and adipogenic cell lines have generated conflicting results regarding the role of retinoids on osteoblast differentiation, to some extent depending on the concentration of retinoid used. At nanomolar concentrations, ATRA has been reported by several studies to inhibit osteoblastic differentiation and functions, whereas at micromolar concentrations, stimulatory effects have been observed (Figure 4). In fetal rat calvarial cells treated with ATRA at 1 nM or higher concentrations, alkaline phosphatase activity, *Bglap* mRNA, and bone noduli mineralization is inhibited (88, 89). Similar findings have been observed using the human cell line SV-HFO in which dexamethasone-induced osteoblastic differentiation was inhibited by ATRA at 100 nM as assessed by alkaline phosphatase activity, bone noduli mineralization, and increased osteocalcin protein secretion (90). The inhibition of alkaline phosphatase and mineralization seemed to be dependent on RARα/RARβ, whereas osteocalcin secretion was due to activation of RARγ. Inhibition of bone noduli mineralization at osteogenic conditions (ascorbic acid and β-glycerophosphate added) and when osteoblastic differentiation was forced with BMP2 was also observed using the mouse osteoblastic cell line MC3T3-E1 treated with either ATRA, 9-*cis* retinoic acid, or Ro 13-6298 at 1, 10, and 100 nM (62). In this study, the retinoids did not inhibit alkaline phosphatase activity but affected the morphology of the cells, suggesting that the inhibitory effect on mineralization was not primarily due to inhibition of bone formation. Recently, Lind et al. reported that ATRA at 4 and 400 nM inhibited bone noduli mineralization in both primary human osteoblasts and MC3T3-E1 cell cultures (74). The effect in the MC3T3-E1 cell line was associated with decreased cell number and mRNA expression of *Alpl, Bglap, Runx2*, and *Sp7*. The mRNA expression of the ATRA-degrading enzyme *Cyp26b1* is enhanced by ATRA in MC3T3-E1 cells and increasing the endogenous intracellular ATRA levels by the Cyp26 inhibitor R115866 results in decreased mineralization in primary human osteoblasts and MC3T3-E1 cultures, similar to addition of ATRA. In organ-cultured mouse calvarial bones, we have found ATRA (100 nM) to inhibit the expression of a variety of genes associated with both osteoblast differentiation and bone matrix biosynthesis such as *Runx2, Sp7, Alpl, Bglap*, and *Col1a1* (encoding collagen type I, alpha 1) (73).

Whereas retinoids at lower concentrations seem to inhibit osteoblast differentiation, the opposite is generally observed when cells are treated with high, supra-physiological concentrations. An early observation was that treatment of the rat preosteoblast cell line UMR-201-10B with 1 μM of ATRA resulted in increased alkaline phosphatase activity and mRNA expression of *Mgp* (encoding matrix gla protein) and *Col1a1*, effects that were synergistically potentiated by 1,25(OH)_2_-vitamin D3 (91). Later on, it was found that treatment of the mouse mesenchymal progenitor cell line C3H10T1/2 with 1 μM ATRA enhances alkaline phosphatase activity, stimulates mRNA expression of *Alpl, Ibsp* (encoding integrin-binding sialoprotein or bone sialoprotein), and *Runx2* and promotes bone noduli mineralization (92–94). Surprisingly, ATRA did not affect *Bglap* or *Sp7* expression (94). When the ATRA concentration was increased to 5 μM in the C3H10T1/2 cell line, alkaline phosphatase activity and *Alpl* and *Bglap* expression were still increased, but no effect on mineralization was observed (95). The stimulatory effects in the C3H10T1/2 cell line were mediated by RARα/RARγ. Enhanced *Runx2* expression is due to ATRA displacing the repressor CCAAT/enhancer binding protein β (C/EBPβ) from the *Runx2* promoter (93). Smad3 is also induced by ATRA in C3H10T1/2 cells and found to be important for the displacement of C/EBPβ, increased *Runx2* expression, and osteoblast differentiation (96). In agreement with these observations, treatment of primary rat calvarial osteoblast cultures with 10 μM ATRA inhibits cell proliferation and stimulates alkaline phosphatase activity and bone noduli mineralization (97). In contrast to findings suggesting that high concentrations of ATRA enhance osteoblast differentiation, it has been reported that ATRA (5 and 10 μM) inhibits mineralization, alkaline phosphatase activity, collagen type I protein, and mRNA expression of *Alpl, Bglap, Col1a* in primary mouse osteoblasts and MC3T3-E1 cells (98).

In the human adipose-derived adult stromal cells (ADAS), 2.5 μM ATRA stimulates alkaline phosphatase activity, the mRNA expression of *Runx2, Bglap*, and *Alpl*, and causes enhanced formation of mineralized nodules, while inhibiting adipocyte differentiation (99). Alkaline phosphatase activity is increased and adipocyte differentiation inhibited by ATRA at 1 μM in the murine preadipocyte cell line 3T3-F442A (100). However, ATRA did not cause complete osteoblast differentiation in 3T3-F442A cells, for *Blap* mRNA expression and bone noduli mineralization were not affected by ATRA.

In the ADAS cell line, ATRA increases the expression of *Bmpr1A* (encoding bone morphogenetic receptor type IA) and addition of BMP2 and ATRA (2.5 μM) synergistically enhance alkaline phosphatase activity, *Runx2* expression, and bone noduli mineralization (99). Synergistic interaction between BMP2 and ATRA (1 μM) on *Alpl* mRNA, but not on *Bglap, Sp7*, and *Ibsp*, expression has also been observed in the C3H10T1/2 cell line (94). Using mouse embryonic fibroblasts (MEF) expressing BMP9 due to adenovirus infection, it has been reported that ATRA (5–20 μM) also synergistically interacts with BMP9 to increase alkaline phosphatase activity, *Bglap* mRNA, osteocalcin protein expression, and bone noduli mineralization (95). Although it seems as if the stimulatory effects by ATRA *per se*, or in combinations with BMPs, can be obtained at micromolar concentrations, it has been observed in the murine preadipocyte cell line 3T3-F442A that nanomolar concentrations of BMP2 and ATRA can synergistically enhance alkaline phosphatase activity, *Runx2, Bglap*, and *Col1a1* expression, and bone noduli mineralization (100).

An interesting aspect of the role of retinoids in osteoblast differentiation comes from reports showing that treatment of primary osteoblasts or MC3T3-E1 cells with micromolar concentrations of ATRA not only inhibits osteoblast differentiation, but promotes differentiation of cells with an osteocytic phenotype (98, 101). Treatment with ATRA (10 μM) changed the morphology of the cells from cuboidal, typical of osteoblasts in culture, to cells with many ramified extensions, similar to osteocytes in culture. In both cell types, ATRA enhanced intracellular sclerostin and fibroblast growth-factor 23 (FGF23) protein. Furthermore, release of FGF23 protein from the MC3T3-E1 cells was increased by ATRA. The mRNA expression of *Sost* (encoding sclerostin) and *Fgf23* was also up-regulated in MC3T3-E1 cells by ATRA. These findings indicate that ATRA may facilitate the differentiation of osteoblasts into cells with an osteocytic phenotype (Figure 4).

Dentin matrix phosphoprotein 1 (DMP1) and phosphate regulating endopeptidase homolog X-linked (PHEX) are also two markers of late osteoblasts/osteocytes (102). *Dmp1* mRNA is enhanced by ATRA in the MC3T3-E1 cells but not regulated in the primary osteoblasts, whereas the opposite was found for *Phex* mRNA. Lind et al. treated MC3T3-E1 cells with ATRA at 400 nM and also observed enhanced *Dmp1* mRNA expression and DMP1 protein in parallel with decreased expression of *Alpl, Runx2*, and *Bglap* (74). Increased DMP1 protein was also demonstrated by immunohistochemistry in osteocytes of rats treated with high dosages of vitamin A. However, other markers of osteocytes such as *Sost, Phex*, and *Fgf23* were decreased by ATRA. It seems as if ATRA might change the differentiation of osteoblasts to an osteocyte-like phenotype although the latter cell type do not share all phenotypes with native osteocytes.

The *in vivo* data indicate that vitamin A inhibits cortical bone formation without affecting trabecular bone formation, at least in rats treated with supra-physiological levels of vitamin A. This observation is in agreement with several observations showing that low concentrations of ATRA inhibit osteoblast differentiation and function *in vitro*. Interestingly, inhibition of osteoblast differentiation seems to be associated with up-regulation of certain osteocyte characteristics. In contrast, ATRA at high concentrations, or co-treatment of ATRA with BMPs, seems to enhance osteoblast differentiation and function.

### Effects by vitamin A on heterotopic bone formation

Recent studies have indicated that retinoids may have a role in heterotopic bone formation, a disabling condition that can be observed in patients after extensive surgery, such as total joint arthroplasty, traumatic injuries, or in severely wounded soldiers. A similar type of excessive bone formation also can be formed in patients with the rare congenital disease *fibrodysplasia ossificans progressiva* (FOP). These patients exhibit an activation mutation in the BMP type I receptor, *ALK2R206H*. In three experimental models of heterotopic bone formation in mice, including transgenic mice with the human FOP mutation and surgically created pouches in the calf muscles of 2-month-old mice, RARγ agonists CD1530 or R667 prevented the formation of heterotopic bone (103). It is not clear, however, how retinoids block this form of pathological, new bone formation and which cells are targeted. The observations, though, warrant further studies in patients.

## Associations between Vitamin A and Bone Mass in Humans

Supplementation of the diet with vitamins is a common occurrence and there is debate over whether increased vitamin A intake might promote skeletal fragility. The pursuit of a healthy lifestyle often includes a diet where many foods contain vitamin A, as well as taking vitamin A supplements. The currently recommended daily allowance (RDA) of vitamin A is 900 μg/day in adult males and 700 μg/day in adult, non-pregnant or non-lactating females. The tolerable upper level (UL) of vitamin A, the highest level likely to pose no ill effects, is suggested to be 3,000 μg/day in adult males and females.

Assessing vitamin A status in individuals is difficult. The most common methods involve determining serum retinol and retinyl ester concentrations. Since vitamin A is stored in the liver and released as needed bound to RBP, measurement of the serum retinol level is not believed to be a sensitive method for determining vitamin A status, except when levels are very low or very high (104). In the case of chronic hypervitaminosis A, measurement of serum retinyl esters have been suggested as an alternative marker (105, 106).

Clinical studies investigating the association between vitamin A and osteoporosis or fracture risk have suggested that vitamin A can be both harmful and beneficial to bone [see Ref. (107) for a more detailed review of the human studies; Table 1]. The studies are mainly observational and as stated above it is difficult to determine vitamin A status in humans. Results can also be influenced by the vitamin D status. Table 1 is a summary of individual studies based on either increases, decreases, or no association of fracture risk or bone mineral density (BMD) to increased vitamin A intake or increased vitamin A intake/low vitamin D. The data suggest that increased vitamin A intake/low vitamin D favors a decrease in BMD and an increase in fracture risk (108–110); however, the effect of increased intake of vitamin A alone appears to be less clear, with increases (25, 26, 111–117), decreases (118–121), and no associations (122–128) to fracture risk and BMD reported. In contrast to the individual observations, a recent meta-analysis of prospective studies has suggested that high retinol intake and blood retinol levels have no effect on total fractures, but significantly increase the risk of hip fracture (129).

**Table 1:** **Human studies evaluating the risk of fractures and BMD to determine the impact of increased vitamin A intake on bone health**.

	Risk of fracture	BMD
**Studies suggesting an association between increased vitamin A intake and osteoporosis or fracture**
JAMA. 287: 47–54, 2002 (25)	↑	
N Engl J Med. 348: 287–94, 2003 (26)	↑	
Am J Epidemiol. 167: 406–11, 2008 (111)		↓
Ann Int Med. 129: 770–8, 1998 (114)	↑	↓
Am J Med. 117: 169–74, 2004 (115)	↑	
J Bone Miner Res. 17: 1349–58, 2002 (117)		↓
**Studies suggesting only a weak relationship, at best, between increased vitamin A intake and osteoporosis or fracture**
Osteoporos Int. 15: 552–9, 2004 (112)	NA (↑)	
Am J Clin Nutr. 79: 155–65, 2004 (113)		NA (↓)
Am J Clin Nutr. 84: 1350–6, 2006 (116)		NA (↓)
**Studies suggesting a beneficial effect of vitamin A for bone health**
J Bone Miner Res. 20: 913–20, 2005 (118)	↓	↑
J Nutr. 125: 1229–37, 1995 (119)		↑
J Clin Endocrinol Metab. 88: 1523–7, 2003 (120)		↑
Bone. 38: 244–8, 2006 (121)		↑
**Studies showing no association of increased vitamin A intake to osteoporosis or fracture**
J Bone Miner Res. 16: 2306–12, 2001 (122)		NA
Osteoporos Int. 14: 418–28, 2003 (123)		NA
Osteoporos Int. 15: 872–80, 2004 (125)	NA	NA
J Clin Epidemiol. 43: 693–9, 1990 (126)	NA	NA
Arch Dermatol. 146: 478–82, 2010 (127)	NA	
Am J Clin Nutr. 82: 581–8, 2005 (128)		NA
**Studies suggesting an association of increased vitamin A intake/low vitamin D with osteoporosis or fracture**
Am J Clin Nutr. 89: 323–30, 2009 (108)	↑	
Arch Osteoporos. 8: 124, 2013 (109)		↓
Clin Biochem. 43: 1064–8, 2010 (110)		↓

*↑, increased; ↓, decreased; NA, not associated*.

Thus, while some studies have suggested that increased vitamin A intake may decrease BMD and promote hip fracture, other studies have not shown increased bone loss or increased fracture risk, and in some instances, protection from bone loss by vitamin A has been suggested. Vitamin D plays a major role in calcium absorption and mineral homeostasis. Vitamin D deficiency is common and some studies have suggested that the risk of osteoporosis and fracture may increase when increased vitamin A intake occurs in individuals with low vitamin D levels (108–110). It is possible an increased risk of osteoporosis and fracture might exist for increased vitamin A intake and/or increased intake in the face of low vitamin D, but it appears that additional *in vivo* animal studies and studies in humans to confirm or dispel these posits will be necessary before clearer estimates of risk emerge.

## Summary

It is well established that hypervitaminosis in rodents decreases cortical thickness by increasing the number of periosteal osteoclasts. On the other hand, it is much less clear how vitamin A affects trabecular bone and if vitamin A regulates bone mass by affecting bone formation. Most of the experimental studies are based upon short-term treatments with high concentrations of vitamin A. There is a need for experiments testing clinically relevant concentrations of vitamin A in long-term studies, where effects on bone mass and activities of osteoclasts and osteoblasts are assessed in both cortical and trabecular bone. Since DXA measurements of BMD in humans do not distinguish between cortical and trabecular bone, there is also a need for prospective clinical studies where vitamin A intake and serum levels of retinoids are related to measurements using peripheral quantitative computed tomography analysis of cortical and trabecular bone. These studies should also include analysis of circulating levels of vitamin D, since the possibility exists that it is the ratio between vitamin A and D which is important for bone mass rather than vitamin A itself.

## Conflict of Interest Statement

The authors declare that the research was conducted in the absence of any commercial or financial relationships that could be construed as a potential conflict of interest.

## Abbreviations

*Acp5*, gene encoding tartrate-resistant acid phosphatase; ADAS, adipose-derived adult stromal cells; *Alpl*, gene encoding alkaline phosphatase; AP-1, activator protein-1; ATRA, all-*trans*-retinoic acid; BCMO1, β-caroten-15,15′-monooxygenase; *Bglap*, gene encoding osteocalcin; BMD, bone mineral density; BMP2, bone morphogenetic protein 2; *Bmpr1A*, gene encoding bone morphogenetic protein receptor 1A; *Calcr*, gene encoding calcitonin receptor; CBP, CREB-binding protein; *Col1a1*, gene encoding collagen type I, alpha 1; CRABP, cellular retinoic acid-binding protein; C/EBPβ, CCAAT/enhancer binding protein β; CRBP, cellular retinol-binding protein; CRE, cyclic AMP response element; CREB, cyclic AMP response element-binding protein; *Csf1r*, gene encoding colony-stimulating factor 1 receptor; *Ctsk*, gene encoding cathepsin K; DMP1, dentin matrix phosphoprotein 1; FABP, fatty acid-binding protein; FGF23, fibroblast growth-factor 23; FOP, fibrodysplasia ossificans progressiva; Fos, FBJ osteosarcoma oncogene; HDAC, histone deacetylase; *Hifa*, gene encoding hypoxia-inducible alpha; *Ibsp5*, gene encoding integrin-binding sialoprotein or bone sialoprotein; IRF-8, interferon regulatory factor-8; MEF, mouse embryonic fibroblasts; *Mgp*, gene encodingmatrix gla protein; *Mitf*, gene encoding microphthalmia-associated transcription factor f; *Mmp2*, gene encoding matrix metallopeptidase 2; NCor, nuclear receptor corepressor; NFATC1, nuclear factor of activated T cells; NF-κB, nuclear factor kappa B; OPG, osteoprotegerin; PHEX, phosphate regulating endopeptidase homolog X-linked; PPAR, peroxisome proliferator-activated receptor; PPRE, PPAR response element; PTH, parathyroid hormone; RALD, retinal aldehyde; RALDH, retinal dehydrogenase; RANK, receptor-activated nuclear factor kappa B; RANKL, RANK ligand; RAR, retinoic acid receptor; RARE, retinoic acid response element; RBP, retinol-binding protein; RDA, recommended daily allowance; ROR, retinoid-related orphan receptor; RORE, ROR response element; RXR, retinoid X receptor; SMRT, silencing mediator of RAR and thyroid hormone receptor; *Sost*, gene encoding sclerostin; *Sp7*, gene encoding osterix; SRC, steroid receptor co-activator; STRA6, stimulated by retinoic acid receptor; *Tnfsf11*, gene encoding RANKL; *Tnfrsf11a*, gene encoding RANK; *Tnfrsf11b*, gene encoding OPG; TRAP5b, tartrate-resistant acid phosphatase 5b; *Twist*, gene encoding twist family basic helix-loop-helix transcription factor 1.
